# Nutrition and Inflammation in Older Individuals: Focus on Vitamin D, *n*-3 Polyunsaturated Fatty Acids and Whey Proteins

**DOI:** 10.3390/nu8040186

**Published:** 2016-03-29

**Authors:** Andrea Ticinesi, Tiziana Meschi, Fulvio Lauretani, Giovanna Felis, Fabrizio Franchi, Carlo Pedrolli, Michela Barichella, Giuseppe Benati, Sergio Di Nuzzo, Gian Paolo Ceda, Marcello Maggio

**Affiliations:** 1 Internal Medicine and Critical Subacute Care Unit, Geriatric-Rehabilitation Department, Azienda Ospedaliero-Universitaria di Parma, Via Antonio Gramsci 14, Parma 43126, Italy; andrea.ticinesi@unipr.it (A.T.); tiziana.meschi@unipr.it (T.M.); flauretani@ao.pr.it (F.L.); 2 Department of Clinical and Experimental Medicine, University of Parma, Via Antonio Gramsci 14, Parma 43126, Italy; sergio.dinuzzo@unipr.it (S.D.N.); gianpaolo.ceda@unipr.it (G.P.C.); 3 Department of Biotechnology, University of Verona, Strada Le Grazie 15, Verona 37134, Italy; giovanna.felis@univr.it; 4 Geriatric Unit, “Guglielmo da Saliceto” Hospital, AUSL Piacenza, Via Taverna 49, Piacenza 29121, Italy; f.franchi@ausl.pc.it; 5 Dietetics and Clinical Nutrition Unit, Santa Chiara Hospital, Azienda Provinciale per i Servizi Sanitari Provincia Autonoma di Trento, Largo Medaglie d’Oro 9, Trento 38122, Italy; carlo.pedrolli@apss.tn.it; 6 Parkinson Institute, Azienda Socio-Sanitaria Territoriale “Gaetano Pini”–C.T.O., Via Bignami 1, Milan 20126, Italy; barichella@parkinson.it; 7 Geriatric Unit, Ospedale G.B. Morgagni–L. Pierantoni, Via Carlo Forlanini 34, Forlì 47121, Italy; nutrizione.clinica.fo@auslromagna.it; 8 Clinical Geriatrics Unit, Geriatric-Rehabilitation Department, Azienda Ospedaliero-Universitaria di Parma, Via Antonio Gramsci 14, Parma 43126, Italy

**Keywords:** vitamin D, omega-3, whey, casein, geriatric, inflammatory markers

## Abstract

Chronic activation of the inflammatory response, defined as inflammaging, is the key physio-pathological substrate for anabolic resistance, sarcopenia and frailty in older individuals. Nutrients can theoretically modulate this phenomenon. The underlying molecular mechanisms reducing the synthesis of pro-inflammatory mediators have been elucidated, particularly for vitamin D, *n*-3 polyunsaturated fatty acids (PUFA) and whey proteins. In this paper, we review the current evidence emerging from observational and intervention studies, performed in older individuals, either community-dwelling or hospitalized with acute disease, and evaluating the effects of intake of vitamin D, *n*-3 PUFA and whey proteins on inflammatory markers, such as C-Reactive Protein (CRP), interleukin-1 (IL-1), interleukin-6 (IL-6) and tumor necrosis factor α (TNF-α). After the analysis, we conclude that there is sufficient evidence for an anti-inflammatory effect in aging only for *n*-3 PUFA intake, while the few existing intervention studies do not support a similar activity for vitamin D and whey supplements. There is need in the future of large, high-quality studies testing the effects of combined dietary interventions including the above mentioned nutrients on inflammation and health-related outcomes.

## 1. Introduction: Inflammaging and Its Relationship with Nutrition

In both genders, aging is associated with a significant rise in serum levels of inflammatory markers, independently of comorbidities and cardiovascular risk factors [1]. This state of chronic low-grade inflammation has been defined as inflammaging and, within certain limits, can be beneficial for healthy aging, stimulating normal tissue remodeling [2]. However, in many cases, the combination of active inflammatory state with reduced antioxidant defenses is detrimental for health. Inflammaging is strictly related with immunosenescence, a reduced or altered immune response to antigenic stimuli, which has been demonstrated in both animal and human models [3]. An age-dependent decline in T and B cells, particularly at the level of CD8+ and CD95− virgin cells, and a concurrent increase of Natural Killer (NK) cells are the distinctive features of this process [4].

Interleukin-1β (IL-1β), Tumor Necrosis Factor-α (TNF-α) and Interleukin-6 (IL-6) have been identified as the key players in inflammaging [2,5]. In physiological acute phase response, IL-6 modulates the synthesis of reactants, including C-reactive protein (CRP), and promotes immune cell activation [6]. Aging is associated with an altered trans-signaling of the IL-6 system, with a decline in soluble IL-6 receptors (sIL-6r) and IL-6 inhibitor sgp130 [5,6]. Thus, the increased signaling due to diminished inhibition induces an inappropriate activation of cellular IL-6 receptors, promoting the inflammatory cascade independently of the presence of antigenic stimuli or tissue damage.

These physio-pathological changes have outstanding clinical implications. In a large cohort of elderly community-dwelling subjects, elevated serum concentrations of IL-6 and IL-1RA have been linked to a decline in physical performance during six-year follow-up period [7], and, in another study, with different degrees of physical disability [8]. A recent translational study has demonstrated that telomere attrition may be the genetic substrate linking chronic low-grade inflammation with altered cell function, and thus with reduced muscle performance [9]. In a large cross-sectional population-based study carried out in female community-dwellers aged more than 65 years, IL-6 levels were also independently associated with higher prevalence of frailty [10]. Moreover, inflammaging may be prodromic to the onset of cognitive disability [11] and multimorbidity [12]. All the chronic diseases with a high prevalence in the older population, including cancer, may in fact be linked with altered immune and inflammatory response [13]. Finally, inflammation also impacts survival, contributing with cognitive symptoms, depression and poor physical performance to define a high-risk profile for mortality [14,15].

The role of nutrition in these processes is of great importance. Chronic low-grade inflammation is the major determinant of the “anorexia of aging”, while acute inflammation may contribute to raising energy requirements, thus driving the onset of “disease-related malnutrition” [16]. The resulting anabolic imbalance between nutrient intake and requirement has been associated with the onset of frailty, muscle mass loss, reduction in muscle strength, and functional dependence leading to overt disability. Diminished food intake and increased energy needs create a vicious circle with unfavorable prognostic trajectory [17].

This catabolic state is greatest during critical illness conditions characterized by poor response to nutritional intervention [18]. In older individuals admitted to hospital for acute illness or chronic disease reactivation, inflammation degree has a greater influence on prognosis than nutritional status [19]. More importantly, the low-grade catabolic state present outside the acute phase is strongly related to inflammaging. This phenomenon, defined as “anabolic resistance”, implies suboptimal skeletal muscle protein synthesis in response to physiologic stimuli and is one of the main determinants of sarcopenia [20].

In healthy active elderly men, inflammaging, measured through mild CRP elevations, causes a decrease in aerobic fitness and insulin resistance in skeletal muscle [21], and stimulates protein synthesis after food intake less effectively than in younger men [22]. Hyperphosphorilation of mTOR and its downstream effector S6K1, with consequent downregulation of mTORC1 signaling pathway, may be the molecular substrates involved [23]. In subjects with chronic diseases, and thus with a higher level of “basal” inflammation, these mechanisms are enhanced and may lead to the development of cachexia [24]. Reduced physical activity also contributes to “anabolic resistance”, inducing a vicious circle very difficult to stop [25].

Nutritional intervention can possibly help to hinder “anabolic resistance”. There are two pathways contributing to restore a normal protein synthesis: raising protein/amino acid intake in order to overcome the increased anabolic threshold, and/or increasing the intake of nutrients that have anti-inflammatory properties. The first approach has been followed in several observational studies and clinical trials investigating the role of nutritional interventions in sarcopenia. Surprisingly, most of the existing evidence claims that protein or amino acid supplementation is not able to parallel improve muscle mass, muscle strength and physical performance [26].

The second approach is discussed in the present review, focused on the main nutrients capable to reduce inflammatory status as actors of the endocrine-nutrition network. The crossroad effector linking nutrition with inflammation is insulin growth factor-1 (IGF-1), whose levels are positively modulated by diet and anabolic hormonal systems and negatively influenced by inflammation and oxidative stress [27]. IGF-1 levels tend to decline with aging, in conjunction with the increase in subclinical inflammatory status [28]. Interestingly, in a large female population-based study, Cappola *et al.*, have demonstrated that low levels of IGF-1 and high levels of IL-6 synergistically contribute to mortality and mobility limitations [29].

Molecular studies have shown that many nutrients might be able to modulate systemic inflammation. These include long-chain saturated fatty acids, oleic acid, *n*-3 polyunsaturated fatty acids, vitamin D, magnesium, calcium, whey proteins, caseins and amino acids like cysteine, histidine, glycine and leucine [30]. However, at the present moment sufficient clinical data in older individuals are present only for vitamin D, *n*-3 polyunsaturated fatty acids and whey proteins [31].

## 2. Vitamin D and Inflammation in Elderly Subjects

### 2.1. Epidemiology and Observational Studies

In older individuals, 25-hydroxyvitamin D (25-OH-D) levels decline with age [32]. A recent systematic review has demonstrated that the prevalence of vitamin D insufficiency or deficiency is extremely high across all age groups and geographical areas, with pandemic proportion. In this scenario, the prevalence in older individuals is even higher, approaching 90% [33]. The causes of suboptimal vitamin D status in the elderly have not been completely demonstrated and mostly remain speculative. They may include insufficient exposure to sunlight, poor nutritional intake, chronic diseases, alterations in body composition with relative increase in fat mass, physical and cognitive disability, polypharmacy [34].

Poor vitamin D dietary intake has a very low probability of being the sole contributing factor to inadequate vitamin D status in the elderly. However, in a recent cross-sectional study carried out on 794 Australian community-dwelling men aged ≥75, only 1% of participants met the recommended Nutrient Reference Value (NRV) for vitamin D intake in their age-group [35]. Similarly, daily intake of vitamin D was inadequate, compared to Recommended Dietary Allowance (RDA), in more than 85% of a cohort of 190 Spanish users of a home care nurse program aged ≥65 [36]. Finally, a cohort of sarcopenic older adults proved to have a lower vitamin D intake than controls [37].

In adult healthy subjects, the relationship between 25-OH-D levels and inflammatory markers is controversial. In a cohort of 1381 adults (mean age 59) from the Framingham Offspring Study, Shea *et al.* failed to demonstrate any association between 25-OH-D and CRP or IL-6 [38]. These findings were confirmed by Clendenen *et al.*, in a smaller cohort of female adults (mean age 55) [39]. However, other larger population-based studies carried out in the U.S. and in Germany identified a U-shaped relationship between 25-OH-D and CRP [40,41]. For example, data from National Health and Nutrition Examination Survey (NHANES), including 15167 adults (mean age 46), showed an inverse relationship between 25-OH-D and CRP only for 25-OH-D levels below 21 ng/mL, and a positive relationship above this threshold [40].

The few population-based studies specifically performed in elderly cohorts (Table 1) revealed a different direction in the association between vitamin D and inflammation. In a large study performed in American community-dwellers aged ≥60, 25-OH-D levels below 24 ng/mL were associated with a significantly increased risk of anemia (OR 1.46, 95% CI 1.06–2.02), while subjects with anemia of chronic inflammation had a significantly higher prevalence of vitamin D deficiency than non-anemic subjects (56% *vs.* 33%, *p* = 0.008) [42]. Data from the InCHIANTI Study, a large population-based study carried out in Italy to identify the determinants of an healthy active aging, revealed that 25-OH-D is inversely associated with IL-6 and positively associated with sIL-6r, independently of a list of potential confounders including physical exercise, caloric intake, smoke, bone mineral density and Activities of Daily Living (ADL) [43]. Interestingly, in this study neither other cytokines nor CRP were associated with 25-OH-D at the multivariate statistical model, thus supporting the hypothesis of the centrality of the IL-6 system in inflammaging [5]. The inverse association of CRP with 25-OH-D levels was instead demonstrated as statistically significant in another similar cross-sectional study carried out on 957 Irish community-dwellers aged ≥60. In this study, also the IL-6/IL-10 ratio was inversely and significantly associated with 25-OH-D [44].

These associations may have important clinical implications. In a small cohort of elderly patients with heart failure, both low serum levels of 25-OH-D and high levels of CRP and IL-6 were independently associated with reduced functional performance, measured through 6-min walk test, and higher frailty score [45]. Moreover, females with severe vitamin D deficiency (≤15 ng/mL) at baseline had higher levels of IL-6 throughout the whole course of recovery after hip fracture than females with 25-OH-D >15 ng/mL [46]. This may imply a worse tissue repair with poorer functional outcomes. The association of a suboptimal vitamin D status with inflammation in older individuals can also determine vascular endothelial dysfunction. This hypothesis has been partly confirmed in studies that have measured endothelial function through serum asymmetric dimethylarginine levels [47], brachial artery flow-mediated dilation [48] and endothelium-dependent and endothelium-independent vasodilation [49]. A poor vitamin D status was also associated with a greater muscle mass loss, detected by dual X-ray absorptiometry (DEXA), in a cohort of Chinese subjects prospectively followed-up for six years, but this association was independent of inflammatory markers [50].

### 2.2. Intervention Studies

The role of vitamin D in modulating inflammation has been studied also in a large number of intervention studies. However, most of them were focused on adult subjects with specific diseases, including asthma, COPD, diabetes, obesity, chronic kidney disease (CKD) under dialytic treatment, and sepsis [51]. These studies gave conflicting results, although they may have been biased by poor generalizability due to extremely specific clinical contexts, low sample sizes, different doses of administered vitamin D, and different outcomes (determination of serum levels of CRP *vs.* other cytokines) [51]. For example, in two randomized controlled trials (RCTs), carried out in adult diabetic patients, supplementation with vitamin D and calcium was associated with a significant decrease in serum levels of CRP, IL-6, IL-1β and TNF-α [52,53]. Similar results were obtained in one RCT carried out in young overweight women with polycystic ovary syndrome [54]. In subjects with non-allergic asthma, vitamin D supplementation improved local eosinophilic airway inflammation [55], while had no clinical effects on disease activity in COPD [56]. Similarly, an observational prospective study conducted in subjects with CKD under dialysis confirmed the role of cholecalciferol supplementation in lowering serum CRP [57]. However, in a RCT performed in a cohort with similar characteristics, the treatment did not result in modulation of alloimmunity and inflammation [58]. Finally, treatment with different doses of cholecalciferol in a large cohort of healthy adult African Americans was not associated with variations of inflammatory markers, although an inverse relationship between 25-OH-D and CRP was detected at baseline [59].

As shown in Table 1, the level of evidence in older individuals is, if possible, even more scarce and contradictory, due to the absence of large randomized controlled trials. The only existing fair-quality study was carried out in a cohort of 613 Australian community-dwellers aged between 60 and 85, who were randomized to receive 1500 µg *vs.* 750 µg cholecalciferol *vs.* placebo monthly for one year. At the end of the follow-up period, serum levels of CRP, IL-6, IL-10, leptin and adiponectin were not statistically different among the three groups, although the 75th percentile IL-6 level was significantly higher in the 1500 µg monthly cholecalciferol group compared with the placebo group (11 pg/mL *vs.* 8.2 pg/mL) [60]. This finding is in contrast with the hypothesized anti-inflammatory properties of vitamin D and instead consistent with the results coming from observational population-based studies. All these data suggest that the relationship between vitamin D and inflammatory markers is U-shaped, and allow to hypothesize that high serum levels of 25-OH-D may be detrimental for health [40,41].

In another RCT, conducted in a cohort of 218 bedridden long-term hospitalized subjects, with a mean age of 84.5 ± 7.5, the supplementation with different doses of cholecalciferol did not influence acute phase reactants, including CRP and fibrinogen, despite normalization of serum 25-OH-D levels [61]. However, the presence of mobility limitations, frailty, multimorbidity and polypharmacy may have acted as strong confounders in this setting.

Similar results were obtained in a cohort of 105 elderly subjects (mean age 78) with congestive heart failure and hypovitaminosis D, where oral ergocalciferol supplementation was associated with significant variations neither in serum TNF-α levels nor in functional outcomes after 10 weeks [62]. Interestingly, in another study carried out on adult patients with the same disease, vitamin D supplementation significantly prevented TNF-α rise during the disease course [63].

Up to date, the only intervention study demonstrating an anti-inflammatory effect of vitamin D supplementation in older age is a small randomized controlled trial carried out in 40 Brazilian female community-dwellers. In this study, the administration of a single dose of 200,000 IU of cholecalciferol resulted in a significant decrease of high-sensitivity CRP levels after four weeks. Interestingly, the decrease was more pronounced in those subjects with the BsmI polymorphism of Vitamin D Receptor (VDR) [64].

### 2.3. Vitamin D and Inflammation in Aging: Mechanisms and Conclusive Remarks

The pleiotropic actions of vitamin D on human health in older age have been appreciated both in clinical and basic science studies [65]. The molecular anti-inflammatory properties of this hormone are well documented [66]. VDR is constitutively expressed by cells playing a key role in inflammation and immunity, including macrophages, that also have the capacity of converting 25-OH-D into its active metabolite by expressing 1α-hydroxylase. The activation of the VDR in macrophages up-regulates the inhibition of NF-κB (I-κB) and down-regulates the expression of TLR2 and TLR4, resulting in decreased production of TNF-α and induced hyporesponsiveness to antigenic stimulation [67,68]. Vitamin D reduces cytokine secretion through its effects on the NF-κB pathway also in lymphocytes [69] and adipocytes [70], favoring immunomodulation and resolution of chronic inflammation [71]. In murine models, it can also stimulate the production of lymphoid cell lineages with regulatory or anti-inflammatory properties, such as T_reg_ cells [72]. Finally, vitamin D stimulates the production of IGF-1, the key player in the cross road linking nutrition and inflammatory pathways [73].

Despite this large amount of molecular evidence suggesting a key role for vitamin D in modulation of inflammation, at the present moment clinical studies do not support this notion. While in adult subjects observational studies provide conflicting results and intervention studies are focused only on disease-specific settings, the level of clinical evidence linking vitamin D with inflammaging in the elderly is even more cryptic. Large, well-designed observational and intervention studies are needed in the future to clarify how the aging process influences the action of vitamin D on inflammation. These studies should consider potential confounders typical of older individuals, such as mobility limitations, cognitive impairment, alterations in body composition, multimorbidity, and, most of all, the possible presence of acute inflammation. The presence of an acute phase response, due to infection, surgery or injury, may in fact be associated with a transient decrease in serum 25-OH-D levels. In a recent systematic review, Silva and Furlanetto concluded that 25-OH-D may behave as an acute phase reactant, highlighting that the relationship between the vitamin D hormonal system and inflammation may be bidirectional [74]. Future research on the association between hypovitaminosis D and inflammaging should therefore strongly consider this hypothesis.

## 3. *n*-3 Polyunsaturated Fatty Acids and Inflammation in the Elderly

### 3.1. Epidemiology and Observational Studies

The effects of *n*-3 polyunsaturated fatty acids (PUFA) on inflammation in human subjects have been extensively studied from both a clinical and a molecular point of view [75]. However, the role of the aging process as confounder in this relationship has not been consistently elucidated [76].

Few studies have assessed the average dietary intake of *n*-3 PUFA in community-living older individuals. Murphy and colleagues have recently demonstrated that, in a large cohort of American healthy adults and seniors, the dietary consumption of *n*-3 PUFA increases with age and is maximum among subjects aged ≥60, even if the levels of intake remain below the recommended doses for cardiovascular health in more than 70% of participants [77]. Very similar results were obtained in the 1990s in a large sample of the Norwegian population [78] and, more recently, in smaller French [79] and Australian cohorts [80].

In healthy adult subjects, the level of intake of *n*-3 PUFA was inversely correlated with circulating levels of CRP and IL-6 in two cross-sectional population-based studies [81,82]. Interestingly, levels of CRP and IL-6 inversely correlated with the consumption of non-fried fish, but positively correlated with the consumption of fried fish [82], thus indicating that the methods of food cooking and processing may have a relevant influence on how fatty acids modulate the inflammatory status. Pischon and colleagues found that the inverse relationship between *n*-3 PUFA and inflammatory markers depends on the intake of *n*-6 PUFA, with the maximum anti-inflammatory effect detected when the intake of both types of nutrients is high [83]. This is somewhat surprising since *n*-6 PUFA are theoretically able to stimulate the arachidonic acid synthesis, leading to pro-inflammatory prostaglandin release [84]. Finally, a high baseline consumption of *n*-3 PUFA was associated with a reduced risk of death from inflammatory diseases in a 15-year large prospective cohort study [85].

Only a few observational studies have investigated the association between *n*-3 PUFA and inflammatory markers focusing specifically on an elderly population [86,87,88]. They are summarized in Table 2. In a cohort from the InCHIANTI study [86], serum levels of total *n*-3 fatty acids were inversely associated with pro-inflammatory markers (IL-6, IL-1ra, TNF-α) and positively associated with anti-inflammatory markers (IL-10, TGFβ), independently of a list of confounders including cardiovascular comorbidity, drug use, energy and macronutrient intake, serum lipids and smoking. The *n*-6 to *n*-3 PUFA ratio was also independently and inversely associated with the modulatory cytokine IL-10, thus supporting the hypothesis that *n*-6 PUFA have a pro-inflammatory effect. Consistently with these findings, in a smaller cohort of Spanish seniors with COPD, TNF-α and IL-6 proved to be positively associated the level of *n*-6 PUFA dietary intake, but negatively associated with the *n*-3 intake [87]. Finally, Kiecolt-Glaser *et al.*, reported that low serum levels of *n*-3 PUFA were associated with TNF-α, IL-6 and depressive symptoms in 43 older community-dwellers [88].

### 3.2. Intervention Studies

The anti-inflammatory role of short- and medium-term *n*-3 PUFA supplementation in adults has been demonstrated only in three small studies. They were carried out on healthy volunteers [89] and critically ill patients with sepsis [90] and pancreatitis [91] admitted to intensive care units (ICU). Conversely, other four RCTs did not support the role of *n*-3 PUFA on inflammatory markers [92,93,94,95]. One of those consisted in a high-quality trial performed on 337 patients with paroxysmal or persistent atrial fibrillation randomized to receive fish oil or placebo for six months [95].

The level of evidence for an anti-inflammatory effect of *n*-3 PUFA supplementation in older individuals is perhaps more solid, as shown in Table 2. In two RCTs carried out on older individuals undergoing hip surgery [96] or admitted to ICU with critical illness [97], the intravenous administration of fish oil-based lipid emulsions for eight days was effective to increase the circulating levels of the anti-inflammatory cytokine IL-10. A decrease in serum levels of IL-8, TNF-α and IL-6 levels was also observed [96,97]. Interestingly, Berger *et al.*, in a RCT enrolling both adult and geriatric patients undergoing cardiopulmonary bypass surgery, showed that *n*-3 PUFA decreased the perioperative inflammation [98].

In a large cohort of healthy Norwegian older individuals living in the community, 2.4 g/day *n*-3 PUFA supplementation for three years was associated with a significant reduction in serum levels of the pro-inflammatory cytokine IL-18, although other inflammatory markers, including CRP, did not change [99]. Similarly, in a smaller cohort of elderly females, 1 g/day *n*-3 PUFA supplementation alone resulted in a significant decrease in TNF-α, while IL-6 and Prostaglandin E_2_ (PGE_2_) decreased only in those subjects where the nutritional intervention was associated with a physical exercise program [100]. Moreover, the supplementation with alpha-linolenic acid, an essential *n*-3 PUFA, combined with a resistance training program, resulted in a reduction of circulating levels of IL-6 after 12 weeks. No changes in other inflammatory cytokines, was observed in a small RCT performed in healthy older subjects [101]. In a cohort of patients with congestive heart failure, *n*-3 PUFA supplementation for three months was associated with a significant decrease in serum IL-6 and TNF-α levels, but not in CRP levels, which actually decreased only in smokers [102]. Conversely, no association between *n*-3 PUFA treatment and inflammation was detected in one RCT conducted in the older age- group of a small cohort of patients with moderate Alzheimer’s disease [103]. However, in this study, the only considered inflammatory marker was PGE_2_.

### 3.3. n-3 PUFA and Inflammation in Aging: Mechanisms and Conclusive Remarks

Other intervention studies performed in elderly subjects focused on translational outcomes and helped to elucidate the mechanisms by which *n*-3 PUFA supplementation may reduce inflammaging. These studies have been recently reviewed by Molfino and colleagues [76]. Briefly, *n*-3 PUFA oral administration has been associated with decreased release of IL-1β and IL-6 from blood mononuclear leukocytes [104], lower response of T lymphocyte proliferation [105,106], reduced activity of lymphocyte particulate phosphodiesterase and glutathione peroxidase [107], NK cells [108], neutrophil respiratory burst and prostaglandin release [109]. Furthermore, *n*-3 PUFA supplementation induced a more favorable serum *n*-6/*n*-3 ratio inducing a leukocyte telomere lengthening that is inversely correlated with IL-6 levels [110]. Reduced cytokine and PGE_2_ release have also been demonstrated after incubating leukocytes with *n*-3 PUFA *in vitro* [75,111].

At a molecular level, the mechanisms by which *n*-3 PUFA modulate inflammatory cytokine production in macrophages may include inhibition of the NF-κB signaling pathway through decreased phosphorylation of the inhibitor I-κB, stimulation of GPR120 (a membrane inhibitor of NF-κB) and stimulation of Peroxisome-Proliferator Activated Receptor gamma (PPAR-γ) receptors that directly control gene activation [75].

In leukocytes, *n*-3 PUFA decreased pro-inflammatory gene activation by altering membrane order, reducing eicosanoid and diacylglycerol synthesis, inhibiting specific isoforms of protein kinase C and mitogen-activated protein kinases, increasing the phosphorylation of phospholipase C-γ, a key signaling enzyme [75]. Many other complex mechanisms in cellular signaling have been postulated in studies investigating the neuroprotective effects of *n*-3 PUFA in Alzheimer’s disease [112], including inhibition of neuro-inflammation [113], although some studies in model animals have given negative results [114]. The link among *n*-3 PUFA, neuro-inflammation and dementia is also supported by observational data from the InCHIANTI study, where low serum *n*-3 PUFA levels have been cross-sectionally associated with both CRP elevation [86] and poor cognitive function [115]. Moreover, the everyday consumption of extra-virgin olive oil, rich in *n*-3 PUFA, seems to be associated to long-term improvement of cognitive outcomes in middle-aged and elderly community-dwellers, through a modulation of neuro-inflammation [116].

In summary, most of the translational and clinical studies support an anti-inflammatory effect of *n*-3 PUFA in older age, although high-quality observational and intervention studies are lacking. The differences in anti-inflammatory effects between acute illness and chronic disease should also be better investigated. It must also be acknowledged that one high-quality RCT aimed at assessing the protective effects of *n*-3 PUFA administration in acute lung injury was stopped because of harmful effects, including excess mortality and days under mechanical ventilation in the intervention group [117]. Thus, the potential risks of *n*-3 PUFA supplementation especially in acute settings should be better investigated.

## 4. Whey Proteins and Inflammation in the Elderly

### 4.1. Biological Role of Whey Proteins between Adulthood and Aging

In elderly subjects, an optimal dietary intake of proteins is of paramount importance for the maintenance of an adequate anabolism in muscle and for prevention of sarcopenia [118]. Thus, the intake recommended for adults (0.8 g/kg/day) is inadequate for preserving muscle mass and functionality in the old age [119]. Inflammaging gives a strong contribution to these phenomena, promoting hypercatabolism and reducing muscle perfusion [120]. At the present moment, the guidelines recommend a protein intake of 1.0 g/kg/day–1.2 g/kg/day in elderly subjects [120,121]. This intake may be even raised to 1.2 g/kg/day–1.5 g/kg/day for those who have an acute disease or undergo physical rehabilitation treatment [120,121]. Despite this, habitual protein intake in older community-dwellers is lower, approaching 0.6 g/kg/day–0.7 g/kg/day, due to early satiety after meals, high prevalence of physical and cognitive disability, and social and financial trouble [120].

Whey proteins account for about 20% of the total protein content of bovine milk, and represent, together with casein, the high-quality fraction of milk proteins. They can be extracted from the liquid byproduct from cheese manufacturing processes [122]. Their high digestibility, quick absorption and elevated content in essential amino acids make whey the ideal nutritional supplement for the aging individual. In fact, whey supplements enhance the physiologic anabolic stimulus to protein synthesis [123] and overcome anabolic resistance more effectively than casein [124] and essential amino acid supplements [125] in older subjects after meals. Beneficial effects of whey supplementation have also been observed after short- and long-term follow-ups in terms of postprandial muscle synthesis [126] and muscle strength [127]. These effects seem to be dose-dependent [128] and are more pronounced when whey supplements have a greater content in the essential amino acid leucine [129]. However, it must be acknowledged that some studies have also given negative results about the favorable biological properties of whey supplements in the elderly. According to these studies, they do not stimulate protein anabolism more effectively than casein or whole milk proteins [130,131], are not associated with better physical performance [132] and are less effective than isocaloric ingestion of amino acid supplements in simulating post-prandial protein synthesis [133].

The putative beneficial effects of whey proteins are due to their favorable composition that allows a quick digestion and absorption, and thus higher concentrations of amino acids in blood immediately after meal [134]. Moreover, they can also effectively stimulate the release of IGF-1 [135], a negative modulator of the inflammatory response [27]. Finally, *in vitro* studies have demonstrated that whey protein extracts can stimulate the NF-κB and MAPK signaling pathways in human neutrophils [136].

Consistently with these mechanisms, several studies have assessed the effect of whey supplementation on inflammatory markers in adult subjects [137]. Most of these studies concluded that it is not associated with a decrease in serum CRP levels in various settings, including individuals with obesity [138,139,140], hypertension [141] and metabolic syndrome [142]. Contrarily, only three small studies conducted in healthy subjects or in patients undergoing minor surgery were consistent with the hypothesized beneficial effects on CRP levels [143,144,145]. Whey proteins were also effective in reducing the exercise-induced release of CRP and IL-6 in young healthy subjects [146].

The results of these studies were recently combined into a meta-analysis by Zhou *et al.*, who summarized that the current state of evidence does not support the hypothesis of an active modulation of inflammation by whey supplements in adult subjects [137]. However, they also concluded that whey supplements have a small, but statistically significant effect in lowering serum CRP in those subjects with baseline values ≥3 mg/L [137]. Thus, from a theoretic point of view, these conclusions may imply that in elderly subjects, where CRP serum levels are persistently above this threshold, whey proteins can exert a beneficial effect on inflammation.

### 4.2. Intervention Studies Focused on the Older Age

There are only four RCTs that have evaluated the role of whey supplementation on inflammatory markers in the elderly [147,148,149,150]. Their essential features are outlined in Table 3. One of them was carried out in healthy active subjects [147], two in subjects with stable COPD [148,149] and one in hospitalized patients with acute ischemic stroke and dysphagia [150]. Overall, two of them supported the hypothesis of an anti-inflammatory effect of whey supplements [149,150], while the others gave negative results [147,148].

All these studies were biased by small sample sizes (in the largest one [147], only 40 subjects were enrolled). The composition of nutritional supplements administered to intervention (*i.e.*, active pressurized whey, whey extracts, and enteral formulas) and control groups (bovine colostrum, casein, and placebo) was not homogeneous. Moreover, whey supplements were prescribed in association with physical training, except for the study performed on patients with acute stroke [150]. Finally, in one study [147], the mean age of participants was 59, despite their definition as “older” individuals. All these elements represent a strong limitation to the validity of the results, and do not allow drawing any conclusive recommendation.

However, another recent fair-quality study suggests that the quality of ingested proteins seems to have a strong modulatory effect on inflammatory markers in the elderly. In this cluster randomized controlled trial, 100 females dwelling in self-care retirement villages and aged between 60 and 90 were randomized to receive progressive resistance training and 160 g of lean red meet six days per week *vs.* progressive resistance training alone. After four months, the intervention group experienced a significant decrease in serum IL-6 levels and a mild, though statistically significant, increase in IGF-1 [151]. The reason for this modulation may depend on the high content in leucine of the employed protein supplement. In fact, other evidence also suggests that leucine may represent the key anti-inflammatory amino acid in proteins [152].

Moreover, an eight-week whey supplementation was associated with a significantly enhanced serological response against 12 out of 14 bacterial types of *Streptococcus pneumoniae* contained in a commercially available vaccine compared with placebo [153]. This study was performed in a very small group (17 subjects) aged on average 67 years, but is in favor of the role of whey supplements in immunomodulation.

In summary, the existing evidence does not support the hypothesis that whey protein has an anti-inflammatory effect in the elderly. However, more high-quality studies are needed to further investigate this possible association.

## 5. Combined Dietary Interventions and Conclusive Remarks

The interaction between different nutrients with putative anti-inflammatory properties on the modulation of inflammation is still poorly investigated. In fact, all the studies reviewed above, linking vitamin D, *n*-3 PUFA or whey proteins with inflammatory markers, considered the effect of only one single nutrient or simple nutritional intervention. Very few studies assessed the effects of combined interventions on inflammation modulation, especially in the elderly population.

However, the hypothesis that the putative anti-inflammatory activity of one nutrient is influenced by the intake of another is supported by some data, and should be considered for designing future studies. For example, Itariu and colleagues interestingly found that the inverse association between vitamin D deficiency and systemic inflammation, measured through IL-6 and CRP in serum, is overcome by treatment with *n*-3 PUFA supplements in severely obese adults, although vitamin D status is unaffected [154]. These results allow to hypothesize that the mechanisms by which vitamin D and *n*-3 PUFA influence inflammation are strictly interconnected, and that the correction of a single nutritional deficiency may be sufficient to limit the negative effects of the other.

In a recent multicenter RCT, a group of 380 sarcopenic older community-dwellers with Short Physical Performance Battery (SPPB, range 0–12) score between 4 and 9 were randomized to receive a vitamin D and leucine-enriched whey protein oral nutritional supplement *vs.* an isocaloric control product. The intervention group showed a significant improvement in anthropometric (appendicular muscle mass), functional (chair stand test) and laboratory parameters (25-OH-D and IGF-1), but unfortunately the effects on inflammation markers are still not available [155]. Ongoing protocols should fill this gap in the future. The RISTOMED study has recently demonstrated that a balanced anti-inflammatory diet, either alone or supplemented with probiotics or d-limonene, modulates inflammatory status in elderly community-dwellers, particularly in those with high baseline CRP levels [156]. Similarly, the NU-AGE study has been specifically designed to investigate the effects of a balanced diet with adequate vitamin D, vitamin B12 and calcium intakes on inflammatory markers, including CRP, IL-1, IL-6 and IL-12 [157]. Moreover, based on the results of the LIFE Study showing that structured physical activity programs are effective to prevent physical frailty in elderly subjects [158], the ongoing SPRINTT project will investigate the combined effects of nutritional supplementation and exercise [159]. Although not specifically designed for evaluating inflammation, this study will help to clarify the mechanisms linking inflammaging, nutrition and physical frailty.

In conclusion, despite basic and translational studies underline the potential role of vitamin D, *n*-3 PUFA and whey proteins as anti-inflammatory nutrients, current state of art of the scientific literature allows to state that only *n*-3 PUFA have a documented, though mild, anti-inflammatory effect in elderly subjects. There is no solid evidence for supporting the anti-inflammatory effects of vitamin D or whey supplements in older individuals. Epigenetic mechanisms have been proposed to significantly influence the association between diet and inflammatory response on an individual basis, and may thus represent one of the reasons of the existing gap between physio-pathological and clinical studies [160]. Gut microbiome and diet-microbiome interactions might also have a role in promoting or controlling inflammation in older persons [13,161]. Future research should better address all these issues, clarifying the molecular and clinical rationale of combined nutritional interventions especially with vitamin D, *n*-3 PUFA and whey proteins.

## Figures and Tables

**Table 1:** Summary of observational (cross-sectional) and intervention (randomized controlled trials) studies exploring the association of vitamin D and inflammatory markers in older individuals.

First Author, Journal, Year [ref]	Country	Study Design	Sample Size	Setting/Health Status	Male (%)	Mean Age (Year)	Mean BMI (Kg/m^2^)	Intervention	Duration (Weeks)	Primary Outcomes	Secondary Outcomes	Results
**Observational studies**												
Perlstein TS, Blood, 2011 [42]	United States	CS	9675	Community-dwelling	43.5	71	-	-	-	Association of vitamin D status with anemia subtypes	-	Vit.D deficiency has a higher prevalence in subjects with chronic diseases and inflammation; OR for anemia in vit.D deficiency 1.46 (95%CI 1.06–2.02)
De Vita F, Age, 2014 [43]	Italy	CS	867	Community-dwelling	43.5	75	27	-	-	Association of serum 25-OH-D levels with hsCRP, IL-1, IL1Ra, Il-10, IL-18, IL-6, sIL6r, sgp130, TNF-α	-	25-OH-D levels are independently and inversely associated with IL-6 and positively with IL6r
Laird E, J Clin Endocrinol Metab, 2014, [44]	Northern Ireland	CS	957	Community-dwelling	50.2	71	29	-	-	Association of serum 25-OH-D with IL-6, TNF-α, IL-10 and CRP	-	Inverse relationship between 25-OH-D and CRP, IL-6 and IL-6/IL-10, CRP/IL-10 and TNF-α/IL-10 ratios
**Intervention studies**												
Waterhouse M, Br J Nutr, 2015 [60]	Australia	RCT	613	Community-dwelling	54	71	27	750 µg *vs.* 1500 µg vit.D_3_ *vs*. placebo monthly	52	CRP, IL-6, IL-10, leptin, adiponectin levels in serum	-	No effect of vit.D_3_ on inflammatory markers
Bjorkman MP, J Nutr Health Aging, 2009 [61]	Finland	RCT	218	Long-term inpatients	18	85	-	1200 IU *vs.* 400 IU vit. D_3_ *vs.* placebo daily	26	25-OH-D, PTH, hsCRP, fibrinogen, markers of bone turnover	-	No effect of vit.D_3_ supplementation on CRP and markers of bone turnover; increase in 25-OH-D and decrease in PTH
Witham MD, Circ Heart Fail, 2010 [62]	United Kingdom	RCT	105	Outpatients with systolic heart failure and vit.D deficiency	65	79	27	100,000 IU vit.D_2_ *vs.* placebo twice (baseline and after 10 weeks)	20	6-min walking distance, QoL, daily activity, functional limitations profile	TNF-α and BNP	Vit.D_2_ treatment did not improve TNF-α concentrations.
de Medeiros Cavalcante IG, Exp Gerontol, 2015 [64]	Brazil	RCT	40	Outpatients with vit.D insufficiency	0	68	28	200,000 IU vit.D_3_ *vs.* placebo once at baseline	4	25-OH-D, PTH, calcium, us-CRP, AGP-A, TAC, MDA	-	Vit.D_3_ megadose administration was associated with a decrease in us-CRP, AGP-A and PTH and an increase in 25-OH-D and TAC.

CS: Cross-Sectional; RCT: Randomized Controlled Trial; BMI: Body Mass Index; IU: International Units; 25-OH-D: 25-hydroxyvitamin D; CRP: C-reactive protein; hs-CRP: high-sensitivity C-reactive protein; us-CRP: ultra-sensitive C-reactive protein; PTH: parathormone; AGP-A: alpha 1-acid glycoprotein; TAC: total antioxidant capacity; MDA: malondialdehyde.

**Table 2:** Summary of observational (cross-sectional) and intervention (randomized controlled trials) studies exploring the association of *n*-3 polyunsaturated fatty acids (PUFA) and inflammatory markers in older individuals.

First Author, Journal, Year [ref]	Country	Study Design	Sample Size	Setting/Health Status	Male (%)	Mean Age (Year)	Mean BMI (Kg/m^2^)	Intervention	Duration (Weeks)	Primary Outcomes	Secondary Outcomes	Results
**Observational studies**												
Ferrucci L, J Clin Endocrinol Metab, 2006 [86]	Italy	CS	1123	Community-dwelling	44.8	68	27	-	-	Association between serum concentrations of fatty acids and IL-6, IL-1ra, IL-10, IL-6r, TNF-α, TGFβ, CRP	-	Total *n*-3 fatty acids are independently associated with lower levels of IL-6, IL-1ra, TNF-α, CRP and higher levels of IL-1ra
de Batlle J, J Nutr Biochem, 2012 [87]	Spain	CS	250	Outpatients with stable COPD	93.6	68	-	-	-	Association between dietary *n*-3 PUFA intake and CRP, IL-6, IL-8, TNF-α	-	Higher intake of α-linolenic acid is associated with lower TNF-α concentrations; higher intake of arachidonic acid is associated with higher IL-6 and CRP concentrations
Kiecolt-Glaser JK, Psychosom Med, 2007 [88]	United States	CS	43	Community-dwellers	41.8	67	-	-	-	Association between serum concentrations of fatty acids, depressive symptoms and TNF-α, IL-6 and sIL-6r	-	Increased serum *n*-6/*n*-3 PUFA ratio is associated with higher TNF-α and IL-6 concentrations
**Intervention studies**												
Gopinath R, Indian J Surg, 2013 [96]	India	RCT	40	Inpatients undergoing hip surgery	60	70	-	Intravenous omega-3 fish oil supplement continuous infusion for 3 days *vs*. placebo	1	Serum CRP, IL-6, IL-8, IL-10	-	Decrease in IL-6 and IL-10 concentrations, increase in IL-8 concentrations, prevention of CRP increase in intervention group
Barros KV, J Parenter Enteral Nutr, 2014 [97]	Brazil	RCT	40	Critically ill patients in ICU	60	71	-	Intravenous fish-oil lipid emulsion 0.2 g/kg of body weight over 6 h for 3 days *vs*. placebo	0.5 (72 h)	Serum IL-1β, IL-2, IL-6, IL-8, IL-10, IL-17, IL-22, TNF-α	-	Lower serum TNF-α and IL-8 concentrations, higher IL-10 concentrations in intervention group
Berger MM, Am J Clin Nutr, 2013 [98]	Switzerland	RCT	28	Patients undergoing elective cardiac surgery	89.2	66	28	Fish oil *vs.* saline infusion 0.2 g/kg 12 h and 2 h before and immediately after surgery	0.2 (1 day)	Serum CRP, IL-6, IL-8, IL-10	Other physiologic and laboratory parameters	Fish oil prevented post-operative increase in IL-6 concentrations
Troseid M, Metab Clin Exp, 2009 [99]	Norway	2 × 2 RCT	563	Community-dwelling	100	70	27	*n*-3 PUFA supplement and/or structured dietary counseling	156 (3 years)	Serum CRP, TNF-α, IL-6, IL-18, adiponectin	BMI and waist circ.	All pro-inflammatory cytokines decreased in intervention groups; IL-18 decreased only in subjects under PUFA
Tartibian B, Nutr Metab, 2011 [100]	Iran	2 × 2 RCT	79	Post-menopausal community-dwelling women	0	62	27	Exercise + Supplement (1 g/day *n*-3 PUFA) *vs.* exercise alone *vs*. supplement alone *vs.* control	24	BMD, markers of osteolysis, TNF-α, IL-6, PGE_2_	-	TNF-α decreases in all groups taking PUFA, while IL-6 and PGE_2_ decrease only for combined intervention
Cornish SM, Appl Physiol Nutr Metab, 2009 [101]	Canada	RCT	51	Healthy active community-dwellers	-	65	-	α-linolenic acid 14 g/day *vs*. placebo while completing a resistance training program	12	Serum TNF-α and IL-6	Muscle strength	Decrease in IL-6 levels in intervention group
Zhao YT, J Int Med Res, 2009 [102]	China	RCT	76	Outpatients with heart failure	73	73	24	*n*-3 PUFA supplement 2 g/day *vs.* placebo	14	Serum CRP, TNF-α, IL-6, intracellular adhesion molecule-1	Serum BNP	Decrease in TNF-α and IL-6, but not CRP, levels in intervention group
Freund-Levi Y, J Alzheimer Dis, 2014 [103]	Sweden	RCT	40	Outpatients with moderate Alzheimer’s disease	-	70	25	PUFA supplement with 1.7 g DHA and 0.6 g EPA *vs*. placebo daily	26	Urinary markers of antioxidant activity, urinary prostaglandins	-	No effect on urinary prostaglandins and antioxidant markers in intervention group

CS: Cross-Sectional; RCT: Randomized Controlled Trial; BMI: Body Mass Index; CRP: C-reactive protein; DHA: docosahexaenoic acid; EPA: eicosapentaenoic acid.

**Table 3:** Summary of intervention (randomized controlled trials) studies exploring the association of whey protein supplements and inflammatory markers in older individuals.

First Author, Journal, Year [ref]	Country	Study Design	Sample Size	Setting/Health Status	Male (%)	Mean Age (Year)	Mean BMI (Kg/m^2^)	Intervention	Duration (Weeks)	Primary Outcomes	Secondary Outcomes	Results
**Intervention studies**												
Duff WR, Int J Sport Nutr Exerc Metab, 2014 [147]	Canada	RCT	40	Community-dwelling	37.5	59	-	Bovine colostrum 60 g/day *vs*. whey protein 38 g/day	8	Muscle strength, antropometric measures, cognitive function	Serum IGF-I and CRP, urinary *N*-telopeptides	No changes in CRP and IGF-1 in both groups
Laviolette L, J Med Food, 2010 [148]	Canada	RCT	22	Outpatients with stable COPD	63.6	65	28	Active pressurized whey supplement *vs*. placebo	16	Muscular strength measures, COPD symptoms	Serum CRP and IL-6	No effect of intervention on inflammatory markers
Sugawara K, Resp Med, 2012 [149]	Japan	RCT	36	Outpatients with stable COPD	93.5	77	-	Whey protein-supplemented oral nutritional supplement 200 Kcal/200 mL per day *vs*. placebo. Exercise in both groups	12	Respiratory functional parameters and serum levels of hs-CRP, IL-6, IL-8 and TNF-α	-	Decrease of serum hs-CRP, IL-8 and TNF-α concentrations in intervention group
de Aguilar Nascimiento JE, Nutrition, 2011 [150]	Brazil	RCT	31	Inpatients with acute ischemic stroke	38.7	74	-	Whey-based *vs.* casein-based enteral nutrition formulas (protein dose 1.2 g/kg/day)	5 days	Serum levels of glutathione peroxidase, CRP and IL-6	-	Decrease in serum IL-6 and prevention of CRP peak in intervention group

CS: Cross-Sectional; RCT: Randomized Controlled Trial; BMI: Body Mass Index; CRP: C-reactive protein; hs-CRP: high-sensitivity C-reactive protein.
